# Prophage identification and molecular analysis in the genomes of *Pseudomonas aeruginosa* strains isolated from critical care patients

**DOI:** 10.1128/msphere.00128-23

**Published:** 2023-06-27

**Authors:** Manuel González de Aledo, Lucia Blasco, Maria Lopez, Concha Ortiz-Cartagena, Inés Bleriot, Olga Pacios, Marta Hernández-García, Rafael Cantón, Maria Tomas

**Affiliations:** 1 Servicio de Microbiología, Hospital Universitario Ramón y Cajal and Instituto Ramón y Cajal de Investigación Sanitaria (IRYCIS); CIBER de Enfermedades Infecciosas (CIBERINFEC), Instituto de Salud Carlos III, Madrid, Spain; 2 Microbiología Traslacional y Multidisciplinar (MicroTM)-Instituto de Investigación Biomédica (INIBIC); Servicio de Microbiología, Hospital A Coruña (CHUAC); Universidad de A Coruña (UDC), A Coruña, Spain; 3 Study Group on Mechanisms of Action and Resistance to Antimicrobials (GEMARA) on behalf of the Spanish Society of Infectious Diseases and Clinical Microbiology (SEIMC), Madrid, Spain; University of Michigan, Ann Arbor, Michigan, USA

**Keywords:** antiviral defense, prophage, CRISPR-Cas, quorum sensing, *Pseudomonas aeruginosa*

## Abstract

Prophages are bacteriophages integrated into the bacterial host’s chromosome. This research aims to analyze and characterize the existing prophages within a collection of 53 *Pseudomonas aeruginosa* strains from intensive care units (ICUs) in Portugal and Spain. A total of 113 prophages were localized in the collection, with 18 of them being present in more than one strain simultaneously. After annotation, five of them were discarded as incomplete, and the 13 remaining prophages were characterized. Of 13, 10 belonged to the siphovirus tail morphology group, 2 to the podovirus tail morphology group, and 1 to the myovirus tail morphology group. All prophages had a length ranging from 20,199 to 63,401 bp and a GC% between 56.2% and 63.6%. The number of open reading frames (ORFs) oscillated between 32 and 88, and in 3/13 prophages, more than 50% of the ORFs had an unknown function. With our findings, we show that prophages are present in the majority of the *P. aeruginosa* strains isolated from Portuguese and Spanish critically ill patients, many of them found in more than one circulating strain at the same time and following a similar clonal distribution pattern. Although a great sum of ORFs had an unknown function, number of proteins in relation to viral defense (anti-CRISPR proteins, toxin/antitoxin modules, proteins against restriction-modification systems) as well as to prophage interference into their host’s quorum sensing system and regulatory cascades were found. This supports the idea that prophages have an influence in bacterial pathogenesis and anti-phage defense.

**IMPORTANCE:**

Despite being known for decades, prophages remain understudied when compared to the lytic phages employed in phage therapy. This research aims to shed some light into the nature, composition, and role of prophages found within a set of circulating strains of *Pseudomas aeruginosa*, with special attention to high-risk clones. Given the fact that prophages can effectively influence bacterial pathogenesis, prophage basic research constitutes a topic of growing interest. Furthermore, the abundance of viral defense and regulatory proteins within prophage genomes detected in this study evidences the importance of characterizing the most frequent prophages in circulating clinical strains and in high-risk clones if phage therapy is to be used.

## INTRODUCTION

*Pseudomonas aeruginosa* is a ubiquitous opportunistic pathogen associated with numerous nosocomial infections, often related with medical devices and procedures (e.g., endovascular catheters, mechanical ventilation or surgical wound, and burn infections) as well as chronic respiratory diseases, such as those present in cystic fibrosis and bronchiectasis patients and those with chronic obstructive pulmonary disease (COPD) (1). These non-fermentative Gram-negative rods are of special concern due to their increasing drug-resistance rates, mainly achieved through a combination of a decreased permeability of the outer membrane, active drug expulsion from the bacterial cell, and the acquisition of mobile genetic elements encoding antibiotic-resistance genes (2). *P. aeruginosa* has also been included into the ESKAPE group, a classification of six bacteria to which special attention has to be paid due to their increased antimicrobial-resistance rates (3). Besides, its ability to produce a wide range of virulence factors such as biofilms, exotoxins, siderophores, or secretion systems makes this pathogen a serious threat able to adapt to a continuously changing environment (4).

On the other hand, bacteriophages or phages are viral particles infecting bacteria and archaea. In recent years, special consideration has been drawn to lytic phages due to their ability to target and eradicate specific clones of a given bacteria, outstanding as promising narrow-spectrum antimicrobial weapons (5). Nevertheless, the importance of prophages—phages integrated into the bacterial host’s chromosome—is still starting to be recognized. These viruses have been considered for years as “dormant” as the majority of their genes are generally repressed. However, they have now been shown to interact with the bacterial cell’s regulatory cascade to interfere with the host’s immune system as well as to encode toxins, lytic proteins, and antimicrobial-related genes (6). However, in spite of being known for decades, it is still a lot what remains unexplored.

Over time, bacteria have evolved and acquired numerous mechanisms for their defense against bacteriophages including restriction-modification (RM) systems, the clustered regularly interspaced short palindromic repeats (CRISPR) and CRISPR-associated (*cas*) genes, the abortive infection (Abi) systems as well as the accumulation of a variety of mutations in surface receptor proteins (7, 8). Interestingly, in the midst of this evolutionary race between bacteria and the viruses that prey on them, toxin/antitoxin (T/A) systems have also been proposed as an anti-phage defense. These modules, consisting of a toxin that arrests cell growth and a cognate antitoxin that neutralizes the toxin, are known to maintain plasmid stability and to confer a persister state of the bacterial host cells, allowing antibiotic tolerance. However, there is growing evidence that some T/A systems may act as anti-phage bacterial defenses (9, 10).

Moreover, new anti-viral mechanisms have been recently described such as the use of cyclic nucleotides as signaling molecules [CBASS (11), Pycsar (12), adenine deamination—RADAR (13)] and NAD+ depletion as a widespread response to viral infection (14, 15).

Besides, in the environment bacteria live in complex, spatially structured, and multispecies communities (16), which highlights the need to consider antiphage strategies at the community level. The mechanisms involved are quorum sensing network (17 - 19), the release of extracellular vesicles (20, 21) or the formation of biofilm structures (16, 22).

Finally, chemical inhibition of phages through small molecules secreted in the extracellular space represents another effective multicellular strategy against phage infection, which, unlike most defense systems described until now, does not rely on proteins or RNA. Among them, we could highlight anthracycline, aminoglycosides, and viperin molecules (23).

On the other hand, bacteriophages have developed counterdefense mechanisms such as anti-CRISPR (Acr) proteins and viral DNA methyltransferases. Acr proteins, firstly discovered in prophages infecting *P. aeruginosa* strains (24), are small peptides (typically between 50 and 150 amino acids) known to inhibit CRISPR-Cas activity by binding the different elements that form the CRISPR machinery, and thus preventing DNA recognition, or by inhibiting Cas proteins’ activity once the protein complex has been assembled around the target DNA (25). Moreover, bacteriophages have been shown to encode DNA methyltransferases while lacking their cognate restriction endonuclease. These enzymes, known as orphan DNA methyltransferases, mimic those of the restriction-modification systems, making the bacterial restriction endonucleases unable to recognize viral DNA as exogenous and impeding its cleavage (26).

The goal of the current work is to broaden knowledge into the nature, composition, and role of the prophages found within a set of a *P. aeruginosa* strain collection recovered from critically ill patients from both Portuguese and Spanish hospitals and to analyze the genes they harbor to overcome bacterial defenses.

## MATERIALS AND METHODS

### Isolate collection and genome sequencing

For the present study, 53 *P*. *aeruginosa* strains were studied. They were recovered from urinary tract, low respiratory tract, and intra-abdominal infections in patients admitted to ICU in both Portuguese (*n* = 40) and Spanish (*n* = 13) hospitals as part of the STEP and SUPERIOR studies (27 - 29). The whole genome extraction and sequencing methods are described elsewhere, and genomes were deposited in GenBank under the Bioproject PRJNA629475 and accession numbers JABDTR000000000-JABDVT000000000. Nucleotide sequence data reported are available in the Third Party Annotation (TPA) Section of the DDBJ/ENA/GenBank databases under the accession numbers TPA: BK061475-BK061480 and BK061585-BK061591.

### Genome assembly and prophage identification

The 150 bp paired-end sequence reads were *de novo* assembled using SPAdes v3.13.0 (https://cab.spbu.ru/software/spades/) with the following settings: minimum contig length 300 bp, minimum contig coverage five, and no read trimming (30).

Assembled genomes of *P. aeruginosa* isolates were analyzed with the Phaster (PHAge Search Tool Enhanced Release) software (https://phaster.ca/) and only those identified as intact (score >90) were included into the study (31). Prophages within the different strains were compared through Nucleotide BLAST v2.13.0 (Basic Local Alignment Search Tool, https://blast.ncbi.nlm.nih.gov/Blast.cgi) and those with a coverage >80% and identity >90% were considered to be the same prophage.

### Viral genome annotation

Prophages found in more than one strain simultaneously were selected for further analysis. Viral genomes were annotated using RAST software v2.0 (Rapid Annotation Using Subsystem Technology, https://rast.nmpdr.org/rast.cgi). In addition, all ORFs were manually annotated with HMMER v3.3.2 (http://hmmer.org/) and HHpred v57c8707149031cc9f8edceba362c71a3762bdbf8 [https://toolkit.tuebingen.mpg.de/tools/hhpred (32)]. For HMMER, annotations were considered valid for E-values below 0.01 and for HHpred for E-values ≤ 10^−5^ (i.e., probability >98%). Whenever discordance between annotations was found, RAST was prioritized to HMMER and HMMER to HHpred.

To establish the tail morphology group, the closest bacteriophage candidate given by Phaster was searched into the Virus-Host database (33). These results were subsequently confirmed by a BLAST search against the NCBI database using the terminase large subunit.

A phylogenetic tree was constructed using the terminase large subunit nucleotide sequence as a reference. Sequences were aligned using MAFFT v7.407 (34) default options, and phylogenetic analysis was performed in RaxmlHPC-PTHREADS-AVX2 v8.2.12 (35) under the GTRGAMMA model and 100 bootstrap replicates. FigTree (http://tree.bio.ed.ac.uk/software/figtree/) was used to visualize the phylogenetic tree.

Furthermore, antibiotic resistance genes were searched in the viral genomes through RGI v5.2.1 (Resistance Gene Identifier, https://card.mcmaster.ca/analyze/rgi) and ResFinder v4.1 (https://cge.cbs.dtu.dk/services/ResFinder/). Anti-CRISPR proteins were also investigated through several tools: CRISPRCasFinder v1.1.2 (https://crisprcas.i2bc.paris-saclay.fr/CrisprCasFinder/Index), AcrFinder (https://bcb.unl.edu/AcrFinder/index.php), PaCRISPR (https://pacrispr.erc.monash.edu/server.jsp), and anti-CRISPRdb (http://guolab.whu.edu.cn/anti-CRISPRdb/search.php).

Prophage integration sites were identified analyzing their flanking genes and locating them in a reference strain (PAO1). When possible, this was confirmed by BLAST analysis using the *attL* and *attR* sequences provided by Phaster for each prophage.

In addition, protein three-dimensional structure was predicted using Phyre2 (Protein Homology/analogY Recognition Engine, v2.0, http://www.sbg.bio.ic.ac.uk/phyre2/html/page.cgi?id=index) from the aminoacidic sequence. This software compares the obtained hidden Markov model with a set of models generated from known protein structures to detect high confidence similarities. Besides, protein three-dimensional structure was also predicted using the Expasy Swiss-Model tool (36).

### Prophage activation, bacteriophage isolation, and transmission electron microscopy

Prophage activation was induced with mitomycin C as described by López et al. (37). For that purpose, *P. aeruginosa* strains were incubated overnight at 37°C with shaking (180 rpm) and were afterward used to inoculate 15 mL of Luria-Bertani (LB) broth. Optical density was measured at a wavelength of 600 nm (OD_600_) until cultures reached 0.5. Then, mitomycin C was added at a concentration of 10 µg/mL and cultures were incubated with the same conditions until they became clear, meaning that lysis had occurred (approximately 1–3 h). Cell debris was then precipitated by centrifugation at 3,500 rpm for 10 min and the supernatant was filtered through a 0.22 µm filter (Millipore Express PES membrane, Merck, Darmstadt, Germany). After addition of NaCl to a final concentration of 0.5 M, suspensions were mixed and left on ice for 1 h. Subsequently, the suspensions were centrifuged at 3,500 rpm for 40 min at 4°C and the supernatants were collected into sterile tubes, to which PEG 6000 (10% wt/vol) was added and dissolved by rocking the tubes at room temperature for 1 h. After an overnight incubation at 4°C, bacteriophages were precipitated at 3,500 rpm for 40 min at 4°C and resuspended in SM buffer (0.1 M NaCl, 1 mM MgSO_4_, 0.2 M Tris-HCl, pH 7.5). Finally, samples were stored at 4°C until preparation for TEM with a JEM-1011 (JEOL, Akishima, Japan) electron microscope.

## RESULTS AND DISCUSSION

### Genome assembly and prophage search

Genomes belonging to the 53 *P*. *aeruginosa* isolates were *de novo* assembled. The number of contigs of the obtained bacterial genomes ranged from 206 to 3,252 (mean 1,633). The number of intact bacteriophages found in each genome ranged from 0 to 5, with a median of 2, adding a total of 113 prophages (Table 1). Among them, 18 prophages were found to be present in more than one strain simultaneously by BLAST analysis. In 7/53 (13.2%) strains, no intact prophages were found.

**TABLE 1:** Information on the analyzed *P. aeruginosa* strains and number of intact prophages found by Phaster^*a*^

Strain	Contigs (*n*°)	Clonal complex	Region (country)	Source^*b*^	Intact phages (*n*°)
1–13	365	235	Aveiro (Portugal)	IAI	3
2–10	2,393	499–1LV	Lisbon (Portugal)	LRTI	3
2–21	2,178	309–1LV	Lisbon (Portugal)	IAI	1
2–29	645	235	Lisbon (Portugal)	UTI	3
3–5	1,871	348–1LV	Lisbon (Portugal)	UTI	1
3–38	533	348	Lisbon (Portugal)	LRTI	2
3–41	1,084	348–1LV	Lisbon (Portugal)	LRTI	1
3–49	709	235	Lisbon (Portugal)	IAI	4
3–58	514	348	Lisbon (Portugal)	IAI	1
3–69	2,676	554–1LV	Lisbon (Portugal)	LRTI	0
4–14	1,846	313–1LV	Coimbra (Portugal)	LRTI	4
4–17	493	235	Coimbra (Portugal)	UTI	4
4–29	2,595	179–1LV	Coimbra (Portugal)	LRTI	1
4–71	346	235	Coimbra (Portugal)	IAI	4
4–79	206	235	Coimbra (Portugal)	UTI	4
4–86	265	235	Coimbra (Portugal)	IAI	4
4–92	387	235	Coimbra (Portugal)	UTI	4
4–93	567	235	Coimbra (Portugal)	UTI	4
4–94	814	235	Coimbra (Portugal)	IAI	4
4–120	247	235	Coimbra (Portugal)	UTI	4
4–121	1,216	235–1LV	Coimbra (Portugal)	UTI	3
4–125	1,807	253–1LV	Coimbra (Portugal)	IAI	0
5–15	936	235	Porto (Portugal)	IAI	1
5–23	449	244	Porto (Portugal)	LRTI	3
6–25	2,029	244–1LV	Porto (Portugal)	LRTI	3
6–38	316	253	Porto (Portugal)	IAI	2
6–59	1,402	179	Porto (Portugal)	UTI	0
6–102	2,552	446–1LV	Porto (Portugal)	LRTI	1
7–41	2,487	3292–1LV	Lisbon (Portugal)	LRTI	1
8–1	2,824	348–1LV	Lisbon (Portugal)	LRTI	0
8–12	2,922	253–1LV	Lisbon (Portugal)	LRTI	0
8–24	876	244–1LV	Lisbon (Portugal)	UTI	4
8–36	798	244	Lisbon (Portugal)	UTI	5
8–58	1,669	244–1LV	Lisbon (Portugal)	IAI	0
9–25	1,248	244–1LV	Lisbon (Portugal)	UTI	3
9–35	3,182	308–1LV	Lisbon (Portugal)	LRTI	0
9–41	1,804	235–1LV	Lisbon (Portugal)	LRTI	1
9–86	331	554	Lisbon (Portugal)	IAI	2
10–58	369	244	Porto (Portugal)	LRTI	4
10–99	2,212	1233–1LV	Porto (Portugal)	IAI	1
C11	1,942	175	Barcelona (Spain)	UTI	3
C58	2,635	175–2LV	Barcelona (Spain)	UTI	2
D4	2,903	27–1LV	Seville (Spain)	IAI	2
E16	2,438	175–1LV	Santander (Spain)	UTI	1
E17	2,525	175–1LV	Santander (Spain)	IAI	1
F43	2,892	175–2LV	A Coruña (Spain)	IAI	1
G6	3,019	175–1LV	Valencia (Spain)	IAI	2
G7	3,252	175–2LV	Valencia (Spain)	IAI	1
G26	2,826	175–1LV	Valencia (Spain)	IAI	2
G31	2,698	175–1LV	Valencia (Spain)	IAI	2
H18	2,573	175–2LV	Majorca (Spain)	UTI	2
H19	2,606	309–2LV	Majorca (Spain)	IAI	2
H52	424	309	Majorca (Spain)	UTI	2
				Total	113

^*a*^ Adapted from Hernández-García et al. (27)

^*b*^ IAI, intraabdominal infection; LRTI, lower respiratory tract infection; UTI, urinary tract infection.

### Prophage analysis and annotation

The resulting 18 prophages were manually annotated by RAST, HMMER, and HHpred. After annotation, five of them were discarded upon realization that they were uncomplete, lacking essential viral proteins.

Among the remaining 13 prophages, phages vB_PaeM-D14A, vB_PaeS-D14B, vB_PaeS-D14C, and vB_PaeS-D14F were present in more than 10/53 *P*. *aeruginosa* isolates. All prophages belonged to the class *Caudoviricetes*, and according to the Virus-Host database, the majority of them (10/13) belonged to the siphovirus tail morphology group. Prophages vB_PaeP-D14I and vB_PaeP-D14S were classified as members of the podovirus tail morphology group, whereas prophage vB_PaeM-D14A was classified as a member of the myovirus tail morphology group. A BLAST search against the NCBI database confirmed these results, showing homology of their terminase large subunit with other viruses from the same group (query cover values of 100.0% and identity values above 90.0%). The only exception was prophage vB_PaeS-D14E, in which those values were 62.0% and 74.6%.

**Fig 1:**
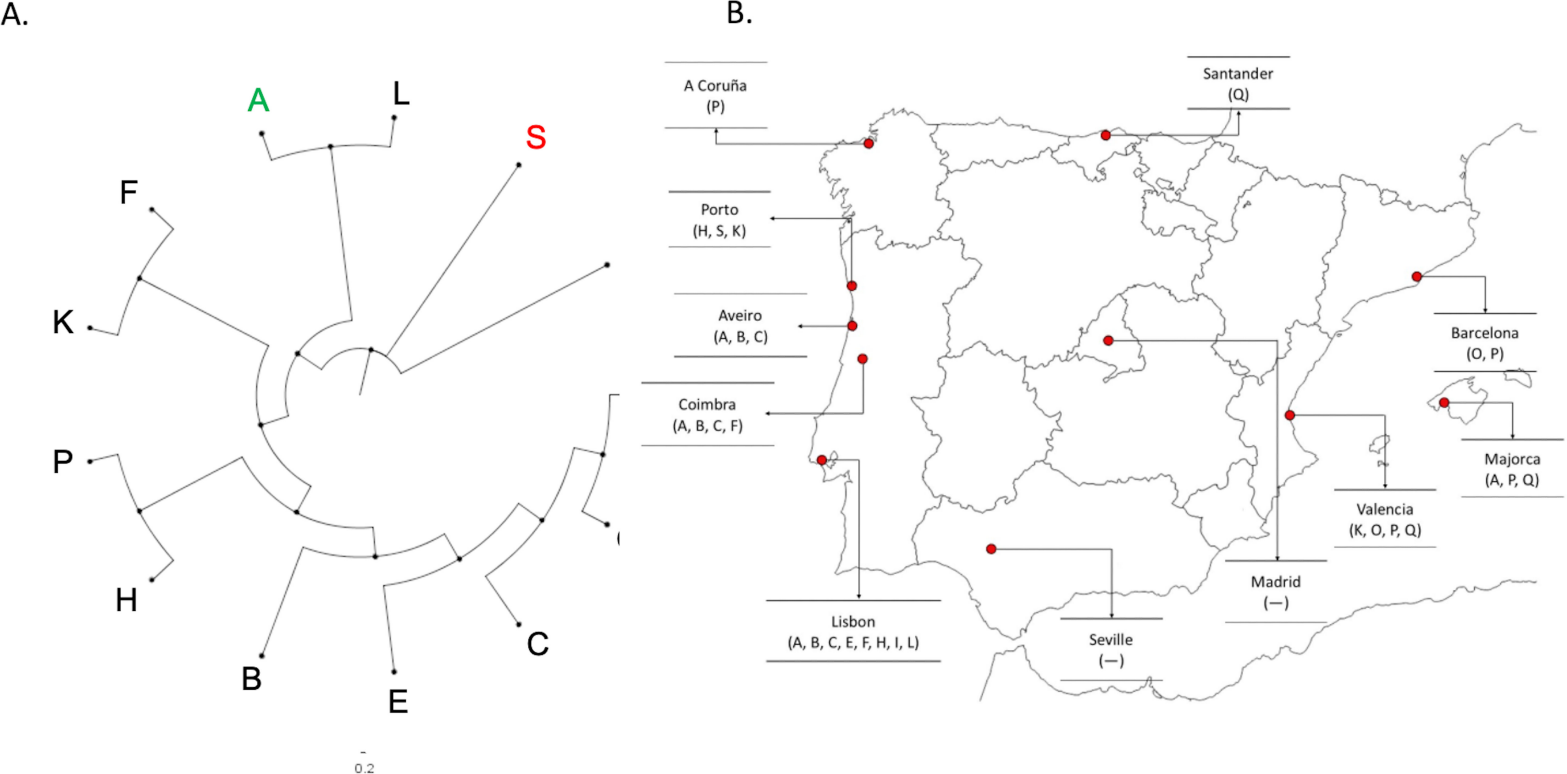
(**A**) Maximum likelihood phylogenetic tree of the 13 prophages studied. Prophages of the siphovirus tail morphology group are represented in black, myovirus in green, and podovirus in red. (**B**) Geographical localization of the prophages in the Iberian Peninsula. A: vB_PaeM-D14A, B: vB_PaeS-D14B, C: vB_PaeS-D14C, E: vB_PaeS-D14E, F: vB_PaeS-D14F, H: vB_PaeS-D14H, I: vB_PaeP-D14I, K: vB_PaeS-D14K, L: vB_PaeS-D14L, O: vB_PaeS-D14O, P: vB_PaeS-D14P, Q: vB_PaeS-D14Q, S: vB_PaeP-D14S. The blank map from the Iberian Peninsula was obtained from https://d-maps.com/carte.php?num_car=2209.

A phylogenetic tree of the 13 prophages was built with the terminase large subunit as a reference (Fig. 1A). It can be noted the close proximity between prophages belonging to the podovirus group and their separation from the rest of the prophage collection. Regarding geographical distribution of the prophages, prophages vB_PaeS-D14O, vB_PaeS-D14P, and vB_PaeS-D14Q were found to be circumscribed to Spanish regions, being the remaining 10 prophages found mainly in Portuguese isolates. Interestingly, despite the geographical proximity to Portugal, prophages found at the A Coruña region resembled more similar to other Spanish regions (Fig. 1B).

Regarding their genome size, all prophages had a length ranging from 20,199 to 63,401 bp, being phage vB_PaeS-D14Q the shortest (20,199–24,677 bp) and phage vB_PaeP-D14I the largest (63,401 bp). Their GC content was found to be between 56.2% and 63.6%, considerably lower than their host’s GC content, which is 65–67% for *P. aeruginosa* (38). The differences in the GC content constitute a sign of an exogenous origin of the prophage regions and usually indicate a recent acquisition (39). The more adapted a prophage is to a species, the more similar its GC content is to their host’s. However, we did not see that prophages with the highest GC content were the most frequent (phages vB_PaeP-D14S and vB_PaeP-D14I were present only in 2/53 strains despite having 63.2–63.8% GC) and neither prophages with the lowest GC content were the least abundant (prophage vB_PaeS-D14C had a GC content of 58.6% and was present in 13/53 strains). In addition, the number of ORFs oscillated between 32 in phage vB_PaeS-D14Q and 88 in phage vB_PaeS-D14O. Finally, regarding ORF annotation, in 3/13 prophages more than 50% of the ORF had an unknown function (Table 2). This is consistent with previous studies (40), highlighting the need to deepen in prophage basic research in order to unravel the different viral mechanisms unknown to date.

**TABLE 2:** Information on the 13 prophages identified in more than one bacterial strain

Prophage	Strains harbouring the prophage (nº)	Tail morfology	Length (bp)	GC content (%)	ORFs (nº)	Annotated ORFs (%)	Accession number	Link accession Genbank
vB_PaeM-D14A	15 (+1*)	Myovirus	36,399–37,203	62.2–63.6	50–52	74.0	BK061475	https://www.ncbi.nlm.nih.gov/nuccore/BK061475
vB_PaeS-D14B	12	Siphovirus	41,283–41,609	61.1	64–65	49.2	BK061476	https://www.ncbi.nlm.nih.gov/nuccore/BK061476
vB_PaeS-D14C	13	Siphovirus	38,595	58.6	60	56.7	BK061477	https://www.ncbi.nlm.nih.gov/nuccore/BK061477
vB_PaeS-D14E	2	Siphovirus	40,769	61.9	62	56.5	BK061478	https://www.ncbi.nlm.nih.gov/nuccore/BK061478
vB_PaeS-D14F	9 (+3*)	Siphovirus	39,504	63.2	57	59.7	BK061479	https://www.ncbi.nlm.nih.gov/nuccore/BK061479
vB_PaeS-D14H	1 (+2*)	Siphovirus	63,196	60.7	81	51.9	BK061480	https://www.ncbi.nlm.nih.gov/nuccore/BK061480
vB_PaeP-D14I	2	Podovirus	63,401	63.8	65	50.8	BK061585	https://www.ncbi.nlm.nih.gov/nuccore/BK061585
vB_PaeS-D14K	2	Siphovirus	35,464–39,623	62.5–62.6	53–56	60.7	BK061586	https://www.ncbi.nlm.nih.gov/nuccore/BK061586
vB_PaeS-D14L	2	Siphovirus	40,814–40,999	61.7	66	48.5	BK061587	https://www.ncbi.nlm.nih.gov/nuccore/BK061587
vB_PaeS-D14O	3	Siphovirus	48,888	56.2	88	46.6	BK061588	https://www.ncbi.nlm.nih.gov/nuccore/BK061588
vB_PaeS-D14P	5 (+3*)	Siphovirus	35,019–39,280	61.1–61.6	48–55	58.2	BK061589	https://www.ncbi.nlm.nih.gov/nuccore/BK061589
vB_PaeS-D14Q	3 (+3*)	Siphovirus	20,199–24,677	58.6–58.9	32–39	71.8	BK061590	https://www.ncbi.nlm.nih.gov/nuccore/BK061590
vB_PaeP-D14S	2	Podovirus	50,727	63.2–63.3	46	50.0	BK061591	https://www.ncbi.nlm.nih.gov/nuccore/BK061591

^*a*^ ORF, open reading frame.

^*b*^ Prophages found fragmented within several contigs.

**Fig 2:**
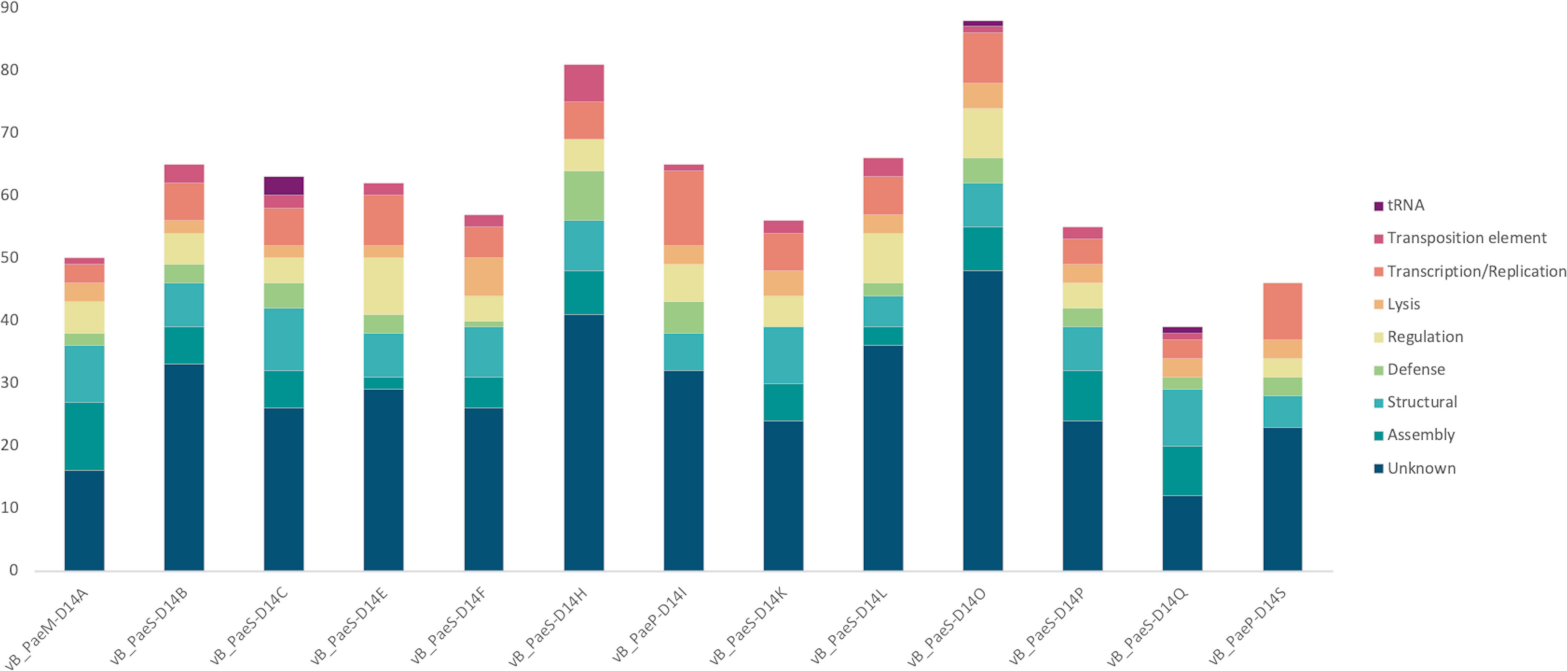
ORF classification in the different analyzed prophages. Y-axis represents the number of ORFs.

When ORF function was classified into different categories, it could be noted that the majority of the genes coded for structural and assembly proteins, viral transcription/replication enzymes, or that they had an unknown function (Fig. 2). However, a number of proteins with special attributes were found in relation to viral defense (anti-CRISPR proteins, toxin/antitoxin modules), prophage interference into their host’s quorum sensing (QS) system, and regulatory proteins.

### Viral defense proteins

#### Anti-CRISPR proteins

Among the 13 analyzed prophages, 11 were found by guilt-by-association to carry putative Acrs through AcrFinder, ranging from one putative Acr in prophage vB_PaeS-D14E to 10 putative Acrs in prophages vB_PaeS-D14F and vB_PaeS-D14O. These proteins were mainly grouped in a single cluster, but in some prophages more than one cluster could be found (two clusters in prophages vB_PaeM-D14A, vB_PaeP-D14I, vB_PaeS-D14K, and vB_PaeP-D14S, and three clusters in prophages vB_PaeS-D14F and vB_PaeS-D14O) (Table 3; Table S1). It should be noted that some predicted Acrs were already annotated with another function (i.e., terminase small subunit, tail structural proteins or holins). However, previous studies propose that some prophage proteins, such as head-tail adaptors or decoration proteins, could simultaneously act as Acr proteins, suggesting that Acr proteins might have evolved from viral structural components (41, 42).

**TABLE 3:** Viral defense and regulatory proteins found in each prophage

	Viral defense proteins						Regulatory proteins	
Prophage	Acr^*a*^	Glycosyltransferases and acetylases	Defense against restriction/modification systems	TA systems	DNA scission proteins	QS	Latency promoting repressors	Other proteins
vB_PaeM-D14A	1 (7)	ND^*b*^	DNA-cytosine methyltransferase	ND	ND	TraR homolog	CII Cro/cI	ND
vB_PaeS-D14B	1 (6)	ND	ND	Putative toxin, BrnT family Putative antitoxin, CopG family	ND	LuxR family protein	Cro CI	ND
vB_PaeS-D14C	1 (2)	Glycosyltransferase family 9	S-adenosyl-L-methionine-dependent methyltransferase	Toxin YafO	ND	TraR homolog	Cro CI CII	ND
vB_PaeS-D14E	0 (4)	ND	DNA-cytosine methyltransferase (EC 2.1.1.37)	Toxin BrnT	ND	LuxR family protein	Cro CI	ND
vB_PaeS-D14F	1 (9)	ND	ND	ND	ND	ND	CII	ND
vB_PaeS-D14H	0 (5)	ND	DNA-cytosine methyltransferase	YefM antitoxin YoeB toxin	Holliday junction resolvase YqaJ-like exonuclease Restriction alleviation protein	ND	Cro CI CII	BCI PrtN
vB_PaeP-D14I	0 (6)	ND	DNA methyltransferase C-5 cytosine-specific DNA methylase	AbiEi antitoxin	Restriction alleviation protein	ND	CI LexA	BCI
vB_PaeS-D14K	0 (9)	ND	ND	ND	ND	ND	Cro/cI CII	ND
vB_PaeS-D14L	1 (4)	ND	ND	YafO family toxin	ND	LuxR family protein	Cro CI	ND
vB_PaeS-D14O	1 (17)	O-antigen acetylase Glycosyltransferase family 9	S-adenosyl-L-methionine-dependent methyltransferase	ND	ND	TraR homolog	Cro CI CII	ND
vB_PaeS-D14P	0 (8)	ND	ND	ND	Holliday junction resolvase	ND	Cro CI CII	BCI
vB_PaeS-D14Q	0 (3)	Glycosyltransferase family 9	S-adenosyl-L-methionine-dependent methyltransferase	ND	ND	ND	ND	ND
vB_PaeP-D14S	1 (6)	ND	Site-specific DNA-methyltransferase	AbiEi antitoxin	ND	ND	CI	BCI

^*a*^ Acr: proven anti-CRISPR proteins. Numbers in brackets refer to putative Acr.

^*b*^ ND, not detected; QS, quorum sensing; TA, toxin/antitoxin.

Besides, the PaCRISPR tool was also used to detect putative Acr proteins (43). All prophages except for one (vB_PaeP-D14S) were found to carry at least one putative Acr, being prophages vB_PaeS-D14O and vB_PaeS-D14P the ones with the greater sum (nine and six, respectively). Unlike the proteins found with the previous tool, putative Acr detected by PaCRISPR did not have a previously known function, being the majority of them (29/36, 80.6%) annotated as hypothetical or unknown phage proteins. Five ORFs were predicted to be a putative Acr simultaneously by AcrFinder and PaCRISPR, and were considered as proven Acr (Table 3).

Finally, two additional Acr proteins were found using anti-CRISPRdb, in prophages vB_PaeS-D14C and vB_PaeP-D14S, both of them showing homology with members of the AcrIIA7 family, with E-values of 3.73e^−30^ and 0.002, respectively. This family of Acr, which has already been characterized in the genomes of tailed bacteriophages, is believed to interfere with the type II-A CRISPR-Cas system by inhibiting Cas9 (44). Given the fact that these ORFs did not have any other assigned function by RAST, HMMER, or HHpred and the considerably high homology scores with known viral defense proteins, Acr could be assigned as their function with high confidence.

#### Defense against restriction-modification systems

Eight out of the 13 prophages coded for DNA methyltransferases, used by the prophage to methylate its own DNA in order to protect it from the host cell’s restriction-modification system, to regulate viral gene expression and to facilitate DNA packaging into the preformed capsids (45, 46). Besides, restriction alleviation proteins were found in prophages vB_PaeS-D14H and vB_PaeP-D14I, known to protect them from the host cell’s restriction-modification system (47, 48) (Table 3).

#### Glycosyltransferases and acetylases

Among the prophages harboring DNA methyltransferases, three of them (vB_PaeS-D14C, vB_PaeS-D14O, and vB_PaeS-D14Q) were found to carry an adjacent glycosyltransferase (Table 3). Bacteriophages are known to encode them to glycosylate their DNA in order to protect it from restriction-modification systems and to modify the O-antigen present in the lipopolysaccharide (LPS) (49). Prophages harness these modifications to avoid the host cell’s superinfection and to prevent the progeny to be retained on the bacterial surface if the lytic cycle is to be initiated. One of these prophages was also found to code for an O-antigen acetylase (vB_PaeS-D14O).

#### Toxin/antitoxin systems

Prophage vB_PaeS-D14B was found to code for a complete toxin/cognate antitoxin module belonging to the type II system with homology to BrnT toxin and a CopG family antitoxin (50). On the other hand, prophage vB_PaeS-D14H coded for the complete type II TA system YoeB/YefM (51). In this same prophage, although two contiguous ORF were firstly annotated as type II TA system YdaT/YdaS homologs, a deep search into literature showed that these proteins were actually the prophage regulatory proteins CII and Cro (52). The type II toxin YafO was also found in prophages vB_PaeS-D14C and vB_PaeS-D14L (53), as well as a type IV antitoxin AbiEi in prophages vB_PaeP-D14I and vB_PaeP-D14S (54) (Table 3). TA systems have been proposed to protect bacteria from phages, together with CRISPR and restriction-modification systems. In this context, it is not surprising to find prophages carrying antitoxins alone, to counteract bacterial defenses, or even toxins alone to compete against external phages preying on their host (9).

#### DNA scission proteins

Prophages coded for junction-resolving enzymes, such as Holliday junction resolvases (in prophages vB_PaeS-D14H and vB_PaeS-D14P) and a YqaJ-like exonuclease (phage vB_PaeS-D14H) (Table 3). These enzymes have been previously described in bacteriophages in the degradation of host’s DNA and in self DNA maturation and cleaving prior to packaging (55).

#### DNA gyrase inhibitor

**Fig 3:**
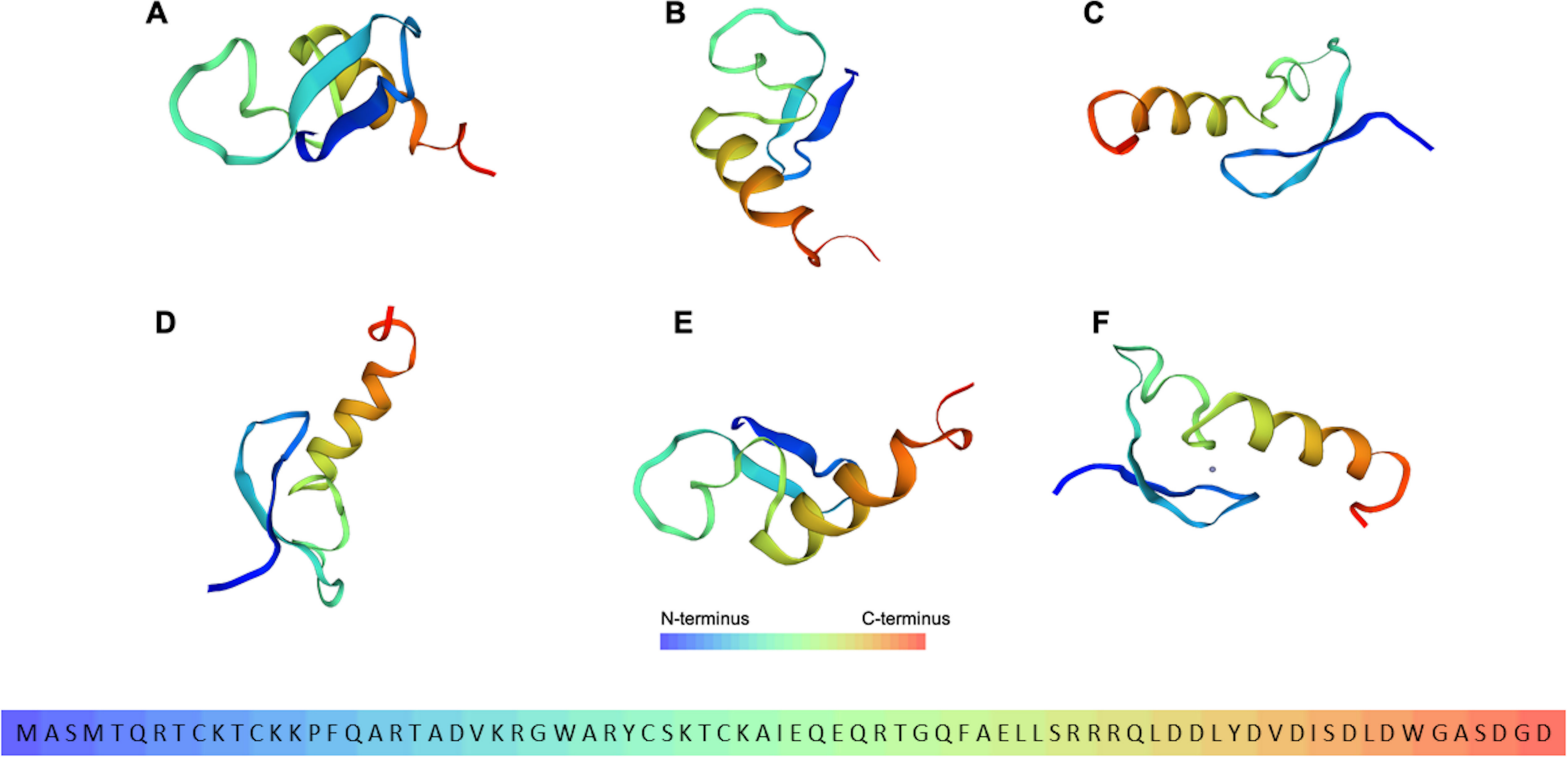
Putative DNA gyrase inhibitor representation as predicted by the Swiss-Model tool by Expasy and its corresponding amino acid sequence. (A through F): different positions of the predicted three-dimensional model. F: purple circle represents an atom of Zn^2+^ as a ligand.

Prophage vB_PaeP-D14I was found to code for a DNA gyrase inhibitor with homology with YacG in *Escherichia coli* (56), as shown by HHpred (>97% probability). This peptide is comprised of 76 amino acids, with a molecular weight of 8.75 kDA and an estimated pI of 8.3. Phyre2 analysis of the aminoacidic sequence yielded a protein model with homology to the above mentioned YacG protein with a confidence of 52.5. Another protein model was predicted by the Swiss-Model tool by Expasy (Fig. 3). This peptide was named as *Pseudomonas* YacG-like DNA gyrase inhibitor. Recently, a peptide with similar anti-DNA gyrase properties has been described for the *P. aeruginosa* bacteriophage LUZ24 (57).

#### Quorum Sensing

Proteins belonging to the LuxR family were identified in prophages vB_PaeS-D14B, vB_PaeS-D14E, and vB_PaeS-D14L. They were present in a single copy in each prophage and did not share any significant similarity with the *P. aeruginosa* QS receptors lasR and rhlR. However, they showed homology with other transcriptional regulators belonging to the LuxR family by both BLAST (>80% query cover and >99% identity) and HMMER (E-value < 1 × 10^−26^). These receptors constitute one of the first and most studied QS systems essential for intercellular communication and gene regulation triggering when a population threshold is reached. Although the archetypical QS tandems consist of a receptor (i*.*e., LuxR) and its cognate autoinducer synthase (i*.*e., LuxI), the presence of LuxR “solos” responsible for intraspecies and interspecies communication has also been described for proteobacteria in general and for *Pseudomonas* in particular (58 - 60). The presence of these receptors in prophage genomes has been linked to a potential role in phages to sense bacterial population density, and therefore to adapt viral infection to it (61) (Table 3). Finally, in prophages vB_PaeM-D14A, vB_PaeS-D14C, and vB_PaeS-D14O, a single copy of a TraR family transcriptional regulator was found in each prophage, all of them conserving a previously described DXXDXA motif in the N-terminal helix. Although they did not share any significant similarity with lasR and rhlR, they showed homology with a *P. aeruginosa* TraR/DksA family transcriptional regulator by BLAST (>85% query cover and >98% identity), HMMER (E-value < 1 × 10^−10^) and HHpred (probability >99%). TraR is a QS receptor, and their homologs have been recently suggested to play a role in redirecting the host’s transcriptional machinery to viral promoters (62, 63).

### Regulatory proteins

#### Lytic/lysogenic cycle switches

In 12 out of the 13 prophages, regulatory proteins in charge of maintaining the lysogenic cycle (CI, CII, and Cro) were found (Table 3). This regulatory network has been characterized in depth for bacteriophage λ, one of the most representative siphovirus. Briefly, the CI repressor is responsible for maintaining a stable lysogenic state by preventing lytic genes’ expression, and for its own synthesis. This synthesis is also stimulated by the CII transcriptional regulator. On the contrary, Cro negatively regulates CII transcription, indirectly reducing CI levels, and thus promoting the lytic cycle. Upon DNA damage, SOS response is triggered and the CI regulator cleaved, consequently initiating the lytic cycle. This process, alongside with the functions of all other regulators (CIII, antitermination protein N and proteins O, P, and Q, among others), have been thoroughly reviewed by Oppenheim and colleagues (64). The fact that no regulatory proteins were found in relation with the lysogenic cycle in prophage vB_PaeS-D14Q responds to a poor annotation of this prophage rather than its absence, given its essential role in prophage homeostasis.

#### Other regulatory proteins

Prophage vB_PaeS-D14H was found to code for the pyocin activating protein PrtN, involved in upregulating pyocin synthesis, a bacteriocin produced by most *Pseudomonas* strains (65, 66). Additionally, the Bacteriophage Control Infection (*bci*) gene, responsible for increasing the host’s infectivity by regulating biofilm production, motility, and virulence factor synthesis in *P. aeruginosa* (67), was found in 4/13 prophages (Table 3).

### Prophage integration sites

**Fig 4:**
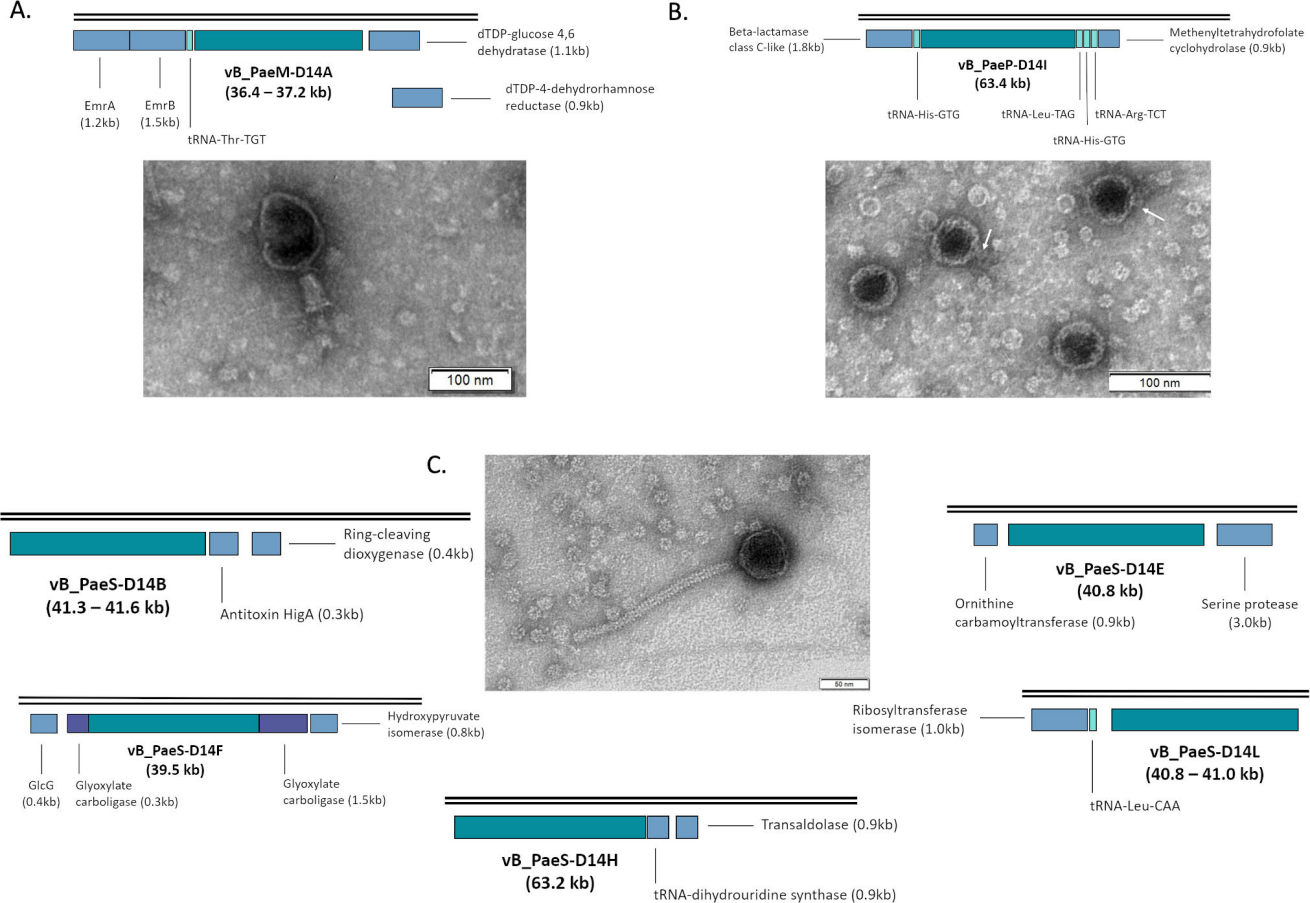
Transmission electron microscopy (TEM) images of the three different phage families belonging to the *Caudoviricetes* class found on this study and representation of their arrangement in the bacterial chromosome. (A) Bacteriophage of the myovirus tail morphology group (vB_PaeM-D14A); (B) bacteriophage of the podovirus group (vB_PaeP-D14I); and (C) bacteriophage of the siphovirus (vB_PaeS-D14B, vB_PaeS-D14E, vB_PaeS-D14F, vB_PaeS-D14H, and vB_PaeS-D14L). White arrows point at viral tails. Sequence segment corresponding to the prophage is not scaled to facilitate visualization.

Successful localization of the prophages’ integration site within the *P. aeruginosa* bacterial chromosome was possible in 7/13 cases (Fig. 4). Prophages vB_PaeM-D14A, vB_PaeP-D14I, and vB_PaeS-D14L were inserted before, between or after host tRNA coding sequences. All tRNA genes were found to be intact, meaning that the prophage insertion did not affect the integrity of the sequence. This is of particular interest in the case of prophage vB_PaeM-D14A, in which the *attR/L* sequence was found to be in the middle of the tRNA-Thr-TGT gene, suggesting that the prophage carried a copy of the same gene so its integration would replace the truncated gene, which is consistent with previous studies (68).

Interestingly, prophage vB_PaeS-D14B was found to be inserted prior to a HigA antitoxin, which was confirmed to be integral by BLAST analysis, meaning that prophage insertion did not disrupt the antitoxin gene. However, the cognate toxin, known to be arranged upstream the antitoxin gene (69), could not be localized within the bacterial genome. On the other hand, prophage vB_PaeS-D14E was localized between a hypothetical protein and an Ornithine carbamoyltransferase (EC 2.1.3.3), both of them conserved and adjacent in PAO1.

Finally, prophage vB_PaeS-D14F was localized integrated into the glyoxylate carboligase (EC 4.1.1.47) gene, disrupting it. This enzyme is responsible for the metabolism of glyoxylate, allowing bacterial growth on glycolate or oxalate. It is also remarkable the finding that this prophage carried a copy of the IclR family transcriptional regulator, involved in the repression of a shortcut in the metabolic pathway of glyoxylate known as the glyoxylate shunt (70). Although the significances of these findings remain unknown, the accumulation of metabolites such as glyoxylate in *P. aeruginosa* has been shown to influence bacterial tolerance and protect cells against antibiotics such as tobramycin through a blockage of the TCA cycle and a reduction in antibiotic uptake (71).

The remaining 5/13 prophages could not be localized within the bacterial genome because the extension of the prophage comprised the whole contig, not being able to identify any flanking ORF in common with the PAO1 or the clonal complexes CC235 and CC175—i.e., the most prevalent in the collection—reference genomes.

### Clonal distribution

Correlation between the *P. aeruginosa* clone and prophage harboring was studied. In the collection of 53 *P*. *aeruginosa* strains, CC235 was found to be the most prevalent (*n* = 14), followed by CC175 (*n* = 10) and CC244 (*n* = 7).

In CC235, prophages vB_PaeM-D14A and vB_PaeS-D14C were found in 13/14, prophage vB_PaeS-D14B in 12/14 and prophage vB_PaeS-D14F in 10/14. The only four pandrug-resistant strains out of the 53, all of them belonging to the CC235, were found to simultaneously harbor phages vB_PaeM-D14A, vB_PaeS-D14B, and vB_PaeS-D14C. Interestingly, the only single strain of the CC235 which did not harbor any of these prophages was also the only CC235 strain which did not carry the GES-13 β-lactamase. This isolate also presented point mutations in genes associated with antimicrobial resistance, such as the membrane porine oprD and the efflux pumps mexY and muxC that the rest of the strains in the clonal complex did not have. Efflux pumps and porines constitute phage receptors, and mutations on these proteins could explain the differential carriage of prophages. However, the number of isolates is not big enough to develop association analyses.

In the strains belonging to the CC175 (*n* = 10); however, more diversity in prophage arrangement was found. Although all of the 10 strains coded for at least one of the prophages under study, the distribution of the phages (vB_PaeS-D14K, vB_PaeS-D14O, vB_PaeS-D14P, and vB_PaeS-D14Q) was uneven. The same appears to happen with CC244, in which prophages vB_PaeS-D14H, vB_PaeP-D14I, vB_PaeS-D14L, and vB_PaeP-D14S were found to be distributed without a clear association (Table 4).

**TABLE 4:** Prophage distribution among the three more frequent sequence types (STs) of the *P. aeruginosa* collection^*a*^

					Antibiotic susceptibility													
**Strain**	**Clonal** **complex**	**O-antigen** **serogroup**	**Prophages**	**β-lactamase**	**P/T^*b*^**	**C/T**	**CAZ**	**FEP**	**ATM**	**IMI**	**MER**	**CIP**	**GM**	**NN**	**AK**	**COL**	**FOS**	**TGC**
1–13	CC235	O11	A, B, C	GES-13	R	R	R	R	R	R	R	R	R	R	R	S	R	R
2–29	CC235	O11	A, B, C	GES-13	R	R	R	R	R	R	R	R	R	R	R	R	R	R
3–49	CC235	O11	A, B, C, F	GES-13	R	R	R	R	R	R	R	R	R	R	R	S	R	R
4–17	CC235	O11	A, B, C, F	GES-13	R	R	R	R	R	R	R	R	R	R	R	S	R	R
4–71	CC235	O11	A, B, C, F	GES-13	R	R	R	R	R	R	R	R	R	R	R	S	R	R
4–79	CC235	O11	A, B, C, F	GES-13	R	R	R	R	R	R	R	R	R	R	R	R	R	R
4–86	CC235	O11	A, B, C, F	GES-13	R	R	R	R	R	R	R	R	R	R	R	S	R	R
4–92	CC235	O11	A, B, C, F	GES-13	R	R	R	R	R	R	R	R	R	R	R	S	R	R
4–93	CC235	O11	A, B, C, F	GES-13	R	R	R	R	R	R	R	R	R	R	R	R	R	R
4–94	CC235	O11	A, B, C, F	GES-13	R	R	R	R	R	R	R	R	R	R	R	S	R	R
4–120	CC235	O11	A, B, C, F	GES-13	R	R	R	R	R	R	R	R	R	R	R	R	R	R
4–121	CC235	O11	A, B, C	GES-13	R	R	R	R	R	R	R	R	R	R	R	S	R	R
5–15	CC235	O11	-	OXA-2	S	S	R	R	R	R	R	R	R	R	I	S	R	R
9–41	CC235	O11	A, C, F	GES-13	R	R	R	R	R	R	R	R	R	R	R	S	R	R
C11	CC175	O4	O, P	-	R	S	R	R	I	R	I	R	R	R	S	S	R	R
C58	CC175	O4/O11	O, P	VIM-2	R	R	R	R	I	R	R	S	R	R	S	S	R	R
E16	CC175	O4	Q	VIM-36	R	S	R	R	I	R	R	R	R	R	S	S	R	R
E17	CC175	O4	Q	-	R	R	R	R	R	R	R	R	R	R	S	S	R	R
F43	CC175	O4/O6	P	-	R	S	R	R	R	R	S	R	R	R	S	S	R	S
G6	CC175	O4	K, P, Q	VIM-20 OXA-2	S	R	R	S	I	R	R	R	R	R	I	S	R	S
G7	CC175	O4	P, Q	VIM-20 OXA-2	S	R	R	R	I	R	R	R	R	R	R	R	R	S
G26	CC175	O4	O, P	-	R	S	R	R	I	R	I	R	R	R	S	S	R	R
G31	CC175	O4	P, Q	VIM-20 OXA-2	S	R	R	R	I	R	R	R	R	R	R	S	R	R
H18	CC175	O4	P, Q	-	R	S	R	R	R	R	R	R	R	R	S	S	R	R
5–23	CC244	O5	H	-	R	S	R	R	R	R	R	R	S	S	S	R	R	R
6–25	CC244	O12	S	-	R	S	R	R	R	R	R	R	R	S	I	R	R	R
8–24	CC244	O5	H, I, L	VIM-2	R	R	R	R	I	R	R	S	R	R	R	S	R	R
8–36	CC244	O5	H, I, L	VIM-2	R	R	R	R	I	R	R	S	R	R	R	S	R	R
8–58	CC244	O5	-	-	S	S	R	R	I	R	R	S	S	S	S	S	R	R
9–25	CC244	O12	S	-	R	S	R	R	R	R	R	R	S	S	I	S	R	R
10–58	CC244	O12	-	-	R	S	R	R	R	R	R	R	S	S	S	S	R	R
3–5	CC348	O12	-	OXA-1	R	S	R	R	R	R	R	R	R	R	R	S	R	R
3–38	CC348	O12	E	OXA-1	R	R	R	R	R	R	R	R	R	R	R	S	R	R
3–41	CC348	O12	E	OXA-1	R	R	R	R	R	R	R	R	R	R	R	S	R	R
3–58	CC348	O12	-	OXA-1	R	R	R	R	R	R	R	R	R	R	R	S	R	R
8–1	CC348	O12	-	-	R	S	R	R	R	R	R	R	R	S	I	S	R	R

^*a*^ A: vB_PaeM-D14A, B: vB_PaeS-D14B, C: vB_PaeS-D14C, E: vB_PaeS-D14E, F: vB_PaeS-D14F, H: vB_PaeS-D14H, I: vB_PaeP-D14I, K: vB_PaeS-D14K, L: vB_PaeS-D14L, O: vB_PaeS-D14O, P: vB_PaeS-D14P, Q: vB_PaeS-D14Q, S: vB_PaeP-D14S. Adaptation from reference (27).

^*b*^ P/T, piperacillin/tazobactam; C/T, ceftolozane/tazobactam; CAZ, ceftazidime; FEP, cefepime; ATM, aztreonam; IMI, imipenem; MER, meropenem; CIP, ciprofloxacin; GM, gentamicin; NN, tobramycin; AK, amikacin; COL, colistin; FOS, Fosfomycin; TGC, tigecycline; S, susceptible; I, susceptible, increased exposure; R, resistant by EUCAST 2020 criteria.

### Prophage isolation and TEM

Finally, prophages detected by *in silico* analysis were proven to be intact and able to initiate the lytic cycle. A representative member of each tail morphology group (myovirus, siphovirus, and podovirus) was chosen to be isolated and imaged by TEM. For the myovirus representative (vB_PaeM-D14A), the *P. aeruginosa* strain 1–13 was selected (Fig. 4A); for the podovirus representative (vB_PaeP-D14I), the *P. aeruginosa* strain 8–24 was selected (Fig. 4B); and for the siphovirus representative (vB_PaeS-D14H/vB_PaeS-D14L), the *P. aeruginosa* strain 8–24 was selected (Fig. 4C).

### Conclusions

This study encompasses the search an analysis of prophages within a set of 53 invasive *P. aeruginosa* clinical strains isolated from critical care patients in different Portuguese and Spanish hospitals. With our findings, we show that these viral entities are present in the majority of circulating strains. Many of the prophages were found in more than one circulating strain simultaneously, following a similar clonal distribution pattern. In only 13.2% of the strains (7/53) no intact prophages—as given by Phaster—were found, showing that prophage harboring is a very frequent trait among circulating *P. aeruginosa* strains in critical care units in Portugal and Spain.

One limitation of our study is that *P. aeruginosa* isolates were sequenced only by short-read bridge amplification (Illumina, Oxford Genomics Centre, Oxford, UK), generating 150 bp fragments which, after assembly, led to 206–3,252 contigs per genome (average of 1,602 contigs/genome). The more fragmented genomes are, the more difficult it is to identify intact prophages, meaning that our search could have missed some of them when split into several contigs. Besides, in 6/13 prophages the viral sequence comprised a whole contig, denoting that the real length could be larger and some ORF could be missing. To circumvent this issue, a combination of both short and long-read sequencing could be performed to obtain high-quality complete bacterial genomes.

Another point of concern is the relatively high proportion of ORF without a known function, being in 3/13 prophages greater than 50%. These findings are aligned with previous studies, which remark not only the vast number of unknown phages sequenced amidst metagenomic data—referred to as viral dark matter—but also the abundance of putative proteins whose function we ignore (72 - 75). In this regard, further studies concerning prophage identification, regulatory pathways, interaction with their host, and protein function should be made.

Although a great sum of viral ORFs is yet to be assigned a function, a number of proteins with interesting roles in altering the host’s regulatory pathways were found within those prophages, supporting the idea that they might influence bacterial pathogenesis. Our study shows the presence of QS-related enzymes (LuxR family proteins and BCI), pyocin synthesis activating proteins (PrtN), and transcriptional regulators such as TraR homologs in almost every prophage under study (12/13), being phage vB_PaeS-D14Q the only exception.

Furthermore, in our work we found putative Acr proteins in every prophage under study, proven Acr proteins in 7/13 prophages and DNA methylation enzymes in 13/13. This highlights the importance of prophage-borne counter-defense mechanisms, which not only protect the prophage against their bacterial host’s immune system but also the host against infection by other phages, enabling its survival and transmission to the bacterial progeny (76, 77). The functions of these proteins and the putative YacG-like DNA gyrase inhibitor should be confirmed experimentally with additional studies.

To continue with, in high-risk clones such as CC235 and CC175, up to four prophages were identified per isolate. These clonal complexes are known for their ability to acquire mobile genetic elements, their elevated antimicrobial resistance rates and their global distribution (78, 79). In particular, CC235, the most prevalent clone among MDR *P. aeruginosa* clinical isolates, has been shown to lack a functional CRISPR-Cas system, thus explaining its ability to acquire exogenous genetic elements such as bacteriophages (80).

Finally, the abundance of lytic cycle regulatory genes, Acr proteins and TA systems within prophage genomes detected in this study evidences the importance of characterizing the most frequent prophages in circulating clinical strains and in high-risk clones if phage therapy is to be used. This way, treatment failure upon the administration of phage therapy related to prophage-borne anti-phage mechanisms could be minimized. Since bacterial isolation and characterization are required prior to the elaboration of a phage cocktail, we propose to include prophage analysis as an additional step. We hope that further studies analyzing prophage profiles in the different circulating clinical strains will shed some light into this issue.

## Data Availability

All data generated or analyzed during this study are included in this published article (and its supplementary information files).

## SUPPLEMENTAL MATERIAL

TABLE S1 msphere.00128-23-s0001.pdf Putative ACR proteins detected.

ASM does not own the copyrights to Supplemental Material that may be linked to, or accessed through, an article. The authors have granted ASM a non-exclusive, world-wide license to publish the Supplemental Material files. Please contact the corresponding author directly for reuse.
