# Supplementary material for: Prophage identification and molecular analysis in the genomes of Pseudomonas aeruginosa strains isolated from critical care patients
Source: mSphere. 2023 Jun 27;8(4):e00128-23. doi: 10.1128/msphere.00128-23 (PMC10449497; doi:10.1128/msphere.00128-23)
Supplement: TABLE S1 — Putative ACR proteins detected. [file msphere.00128-23-s0001.pdf]

Supplementary table S1. Number of putative Acr proteins detected by AcrFinder, Anti-CRISPRdb and PaCRISPR. Results from AcrFinder are represented divided into clusters.

Prophage	*AcrFinder*	*Anti-CRISPRdb*	*PaCRISPR*	*Matches*	*Total Acr*
vB_PaeM-D14A	3+5	0	1	1	8
vB_PaeS-D14B	5	0	3	1	7
vB_PaeS-D14C	0	1	2	0	3
vB_PaeS-D14E	1	0	3	0	4
vB_PaeS-D14F	3+1+5	0	2	1	10
vB_PaeS-D14H	2	0	3	0	5
vB_PaeP-D14I	2+3	0	1	0	6
vB_PaeS-D14K	5+3	0	1	0	9
vB_PaeS-D14L	4	0	2	1	5
vB_PaeS-D14O	5+3+2	0	9	1	18
vB_PaeS-D14P	2	0	6	0	8
vB_PaeS-D14Q	0	0	3	0	3
vB_PaeP-D14S	2+4	1	0	0	7
Total	60	2	36	5	93
